# Precise probiotic therapy: Advances, bottlenecks, and the road to microbiome-informed nutrition

**DOI:** 10.1080/19490976.2026.2623359

**Published:** 2026-01-30

**Authors:** Yuesong Jiang, Shuaiming Jiang, Zhengting Wang, Pengfei Zhu, Jiachao Zhang, Fei Teng, Shi Huang

**Affiliations:** a Faculty of Dentistry, The University of Hong Kong, Hong Kong SAR, People's Republic of China; b School of Food Science and Engineering, Hainan University, Haikou, People's Republic of China; c Single-Cell Center, CAS Key Laboratory of Biofuels, Qingdao Institute of BioEnergy and Bioprocess Technology, Chinese Academy of Sciences, Qingdao, People's Republic of China; d Qingdao Single-Cell Biotech. Co., Ltd, Qingdao, People's Republic of China; e Qingdao Stomatological Hospital Affiliated to Qingdao University, Qingdao, People's Republic of China

**Keywords:** Probiotic heterogeneity, multi-omics integration, food components, precise probiotics, gut microbes, trophic interactions, predictive modeling

## Abstract

The human gut microbiome is a cornerstone of health, yet conventional probiotic therapies often exhibit limited efficacy owing to heterogeneity in host-microbe-environment dynamics. This review dissects the biological and environmental drivers of such variability and highlights emerging frameworks that integrate cross-sectional and longitudinal multi-omics data to predict probiotic treatment outcomes and host metabolic responses. We further spotlight breakthroughs in methodological development in efficient mining and engineering of probiotic strains, which enable the rational design of functionally enhanced, personalized probiotics. By synthesizing these advances, the review underscores the transformative potential of combining data-driven models with precision-engineered microbial therapeutics to address current limitations and usher in a new era of future microbiome-informed nutrition and personalized interventions.

## 1 Introduction

**Figure 1.**
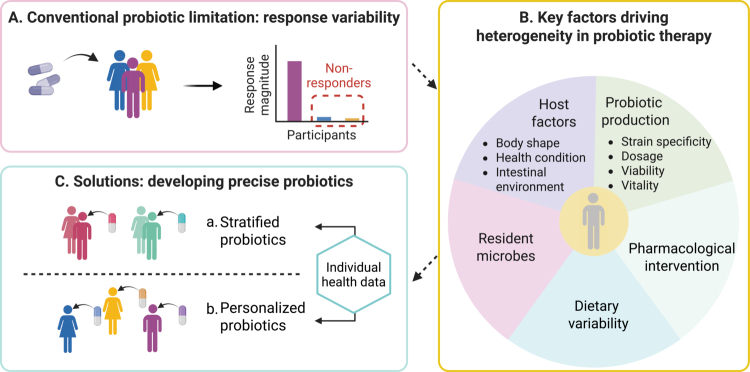
Schematic overview of the factors underlying variability in probiotic efficacy and solutions for precision interventions. A. The variability in probiotic efficacy, characterized by inconsistent response magnitudes and the presence of non-responders among participants, underscores the limitations of conventional probiotic therapies. B. Key factors driving heterogeneity in probiotic outcomes encompass host-specific elements (e.g., body shape, health status, age, intestinal microenvironment), probiotic-related variables (e.g., strain specificity, dosage, viability and vitality), and external influences such as dietary variability and pharmacological intervention. C. The development of precise probiotics, including stratified and personalized formulations tailored to individual health data, has emerged as a promising solution.

The human gut microbiome, a dynamic ecosystem intricately linked to host health, has emerged as a pivotal target for advancing precision medicine and therapeutic interventions.^1^^,^^2^ Among emerging strategies to modulate the microbiome, probiotic treatments—the administration of live microorganisms to confer health benefits—are increasingly applied to improve health outcomes.^3-5^ However, conventional probiotic therapies often demonstrate inconsistent efficacy, with significant variability in response magnitude and a notable proportion of non-responders across populations^6^ (Figure 1A). This heterogeneity arises from a complex interplay of host-specific factors (e.g., genetic makeup, health status, intestinal environment), strain-specific probiotic properties (e.g., genomic variation, metabolic activity),^7^ the composition of resident microorganisms,^8^ and environmental influences (e.g., diet, antibiotics)^9^ (Figure 1B). Such variability underscores the limitations of a “one-size-fits-all” approach and highlights the urgent need for precision in probiotic interventions.^10^

The pronounced heterogeneity and limited predictability of probiotic outcomes highlight the need for a systematic framework that can integrate biological complexity into clinical decision-making. In this context, the paradigm of predictive, preventive, and personalized medicine (3PM) offers a translational foundation to move probiotic interventions beyond empirical trial-and-error approaches by explicitly leveraging inter-individual variation rather than treating it as noise.^11^^,^^12^

Within the 3PM framework, predictive medicine seeks to identify host–microbiome configurations associated with differential probiotic responsiveness using molecular, microbial, and computational biomarkers; preventive medicine emphasizes early, mechanism-informed interventions to reduce disease progression or treatment failure; and personalized medicine focuses on tailoring probiotic strategies to individual biological contexts, including host physiology, microbial ecology, and environmental exposures.^12^ This hierarchy enables a shift from population-averaged efficacy toward stratified and individualized intervention models—an approach particularly well suited to probiotics, whose effects are tightly coupled to host- and microbiome-specific factors.

Recent advances in multi-omics technologies and artificial intelligence are catalyzing a paradigm shift toward precise probiotic therapy. This emerging framework aligns seamlessly with the goals of 3PM paradigm, moving beyond empiricism by integrating individualized host-microbe data to guide the selection of specific probiotic strains, dosages, and formulations. It has given rise to two complementary strategies: (1) stratified probiotics for specific host subpopulations, and (2) personalized probiotics tailored to an individual’s unique microbial and physiological profiles (Figure 1C). A growing body of evidence, summarized in Table 1, demonstrates that probiotic efficacy is highly specific to the strain, the intended use, and the recipient's biological context.

**Table 1.** Representative clinical and mechanistic studies illustrating stratified, personalized, and strain/genotype-specific effects of probiotics.

Category	Strain(s)	Main Findings	Study Type	Ref.
Population-Stratified Effect	*Carnobacterium maltaromaticum*	*C. maltaromaticum* prevents colorectal cancer in females via estrogen-dependent colonization and gut vitamin D–VDR activation.	Mechanistic + Animal model + Human microbiome association	[172]
	*B. bifidum* BGN4 + *B. longum* BORI	Probiotics modulate immune responses in elderly adults in a sex-specific manner, with distinct effects on CD4⁺ T-cell profiles in women and innate immune cells in men.	RCT	[173]
	VSL#3 multi-strain probiotic	VSL#3 significantly reduces microglial activation and TNF-*α* in female but not male AD mice.	Animal model + Mechanistic study	[174]
Personalized Effect	*B. longum* AH1206	AH1206 stably colonizes only individuals with low baseline *B. longum* and lacking specific carbohydrate metabolism genes.	RCT + Mechanistic study	[42]
	Multi-strain probiotic (11 strains)	Multi-strain probiotic colonization and effect on gut mucosa are highly individualized and predictable by baseline microbiome and host features.	RCT + Mechanistic study + Animal model	[10]
	Engineered *E. coli* Nissle 1917	Engineered native EcN significantly reduces colitis severity in mouse models.	Animal model + Mechanistic study + Human microbiome association	[109]
Strain/Genotype Specificity	*L. johnsonii* (NCC 533) + *L. paracasei* (NCC 2461)	Two strains with similar in vitro properties showed different colonization and immune responses in germ-free mice.	Animal model + Mechanistic study	[22]
	*B. longum* transitional clade	A distinct clade of *B. longum*, prevalent in Bangladeshi infants during weaning, utilizes both breast milk and solid food substrates and is associated with infant growth and diarrhea outcomes.	Human microbiome association + Mechanistic study	[23]
	*B. longum* FGDLZ8M1	*B. longum* FGDLZ8M1 most effectively alleviated DSS-induced colitis in mice; this effect was linked to superior gastrointestinal transit tolerance and specific genomic features.	Animal model + Mechanistic study	[175]
	*B. longum* strains carrying the *abfA* gene cluster	Only *B. longum* strains carrying the *abfA* gene cluster effectively alleviate constipation and increase beneficial metabolites.	RCT + Animal model + Mechanistic study + Human microbiome association	[7]
	*B. adolescentis* CCFM 667 + CCFM 669	*B. adolescentis* exhibits strain-specific effects in the alleviation of constipation.	Animal model + Mechanistic study	[176]
	*B. longum + B. infantis + B. bifidum + B. adolescentis + B. breve + B. animalis*	Different species of edible *Bifidobacteria* vary in their effectiveness at relieving constipation.	Animal model + Mechanistic study	[177]
	Various *Bifidobacterium spp.*	FL-SBP (ABC transporter) is essential for fucosyllactose utilization; FL-utilizing *Bifidobacteria* shape infant gut microbiota and metabolites.	Human microbiome association + Mechanistic study	[178]
	Protective *Bifidobacteria*	ABC transporter–dependent acetate production by *Bifidobacteria* protects mice from *E. coli* O157:H7 infection.	Animal model + Mechanistic study	[179]

In this review, we first dissect the fundamental drivers behind the heterogeneity in probiotic efficacy, which stem from a complex interplay of dietary patterns, probiotic strain properties, the resident microbiota, pharmacological interventions, and host factors. We then outline a predictive framework that synergizes cross-sectional and longitudinal data to forecast probiotic efficacy and host metabolic responses under personalized dietary conditions. Finally, we highlight innovative methodological advances in probiotic strain mining and engineering, which are crucial for translating predictions into effective, next-generation probiotics for precise nutrition.

## 2 Fundamental drivers of heterogeneity in probiotic efficacy

The efficacy of probiotics is governed by a complex interplay of host, microbial, and environmental factors that collectively shape a personalized host-microbe landscape. While conventional probiotics often rely on broad, generic mechanisms—such as competitive exclusion or short-chain fatty acid (SCFA) production—their clinical outcomes remain unpredictable due to a frequent mismatch with individual biological contexts. To overcome this limitation, the field is advancing toward precise probiotics that are rationally designed by deconstructing the very sources of heterogeneity. Understanding these factors provides the foundational knowledge for the **predictive diagnostic** pillar of 3PM, enabling the identification of biomarkers and host features that forecast probiotic outcomes. Importantly, probiotic colonization should not be interpreted as a prerequisite for all probiotic-mediated outcomes. Probiotics can act as transient agents, producing bioactive molecules, modulating microbial interactions, or signaling to the host, even without long-term colonization. While stable colonization can prolong some effects, many benefits can arise from short-term presence and repeated use.

These determinants of probiotic success can be categorized into several key dimensions, as explored below:

### 2.1 Dietary variability

Dietary patterns play a crucial role in defining the ecological environment for probiotic colonization within the human gut ecosystem.^13^ Short-term and long-term dietary interventions have been shown to swiftly and significantly modify the gut microbial composition by adjusting substrate availability.^14^ Dietary fibers serve as fermentable substrates that selectively promote probiotic genera such as *Bifidobacteria* and *Lactobacilli* by supporting the microbial synthesis of SCFAs. These SCFAs are essential in maintaining gut balance by reducing luminal pH, inhibiting the colonization of pathogens, and strengthening the integrity of the epithelial barrier through the increased expression of tight junction proteins like occludin.^15^^,^^16^ This principle is leveraged by prebiotics like inulin, fructooligosaccharides, galactooligosaccharides, and resistant starch, which are specifically designed to provide selective substrates for beneficial microorganisms, promoting their growth and enhancing therapeutic outcomes.^5^^,^^17^ For instance, certain oligosaccharides act as competitive inhibitors of pathogen adhesion by mimicking host cell surface glycans, thereby preventing pathogen colonization while promoting probiotic retention.^18^

Beyond substrate support, other dietary components exert more nuanced effects. Diets high in saturated fat can disrupt microbial balance by altering bile acid composition and promoting pro-inflammatory immune responses, thereby creating an intestinal environment that is unfavorable for beneficial microorganisms.^19^^,^^20^ Other dietary components, like polyphenols (e.g., Epigallocatechin gallate), enhance host antioxidant capacity and promote protective SCFA production via microbiota modulation, thereby supporting a favorable intestinal microenvironment for beneficial microorganisms.^21^

### 2.2 Probiotic production: Strain properties, dosage, viability and vitality

The efficacy of probiotic interventions is fundamentally shaped by strain-level intrinsic capabilities, which determine a probiotic’s functional potential and ultimate health benefits. This strain-specificity manifests in divergent colonization patterns, metabolic activities and immunomodulatory effects, even among closely related strains.^22^ Critically, these phenotypic differences are rooted in specific genetic elements, transforming strain selection from an empirical exercise into a rational, target-driven process. For instance, the presence of the *abfA* gene cluster is a key genetic determinant that enables specific *Bifidobacterium longum* strains to utilize arabinan, thereby allowing them to thrive in high-fiber environments and alleviate constipation.^7^ This paradigm, where a precise molecular mechanism explains a targeted health benefit, provides a clear blueprint for probiotic screening and engineering. Beyond metabolic traits, genetic variation underpins host- and population-specific adaptations, as seen in the distinct clade of *B. longum* that thrives during weaning in Bangladeshi children,^23^ as well as specialized therapeutic functions, such as the immune enhancement by *Bifidobacterium bifidum* R-0071^24^ and the mental health benefits linked to *Lactobacillus helveticus* bR0052 and *B. longum* R0175.^25^

Dosage is a critical determinant of probiotic effectiveness. Dose–response effects have been demonstrated in simulated gastrointestinal and colonic fermentation models, where higher probiotic doses resulted in significantly greater strain recovery and measurable increases in metabolic outputs, such as SCFA production, underscoring the importance of adequate dosing for functional probiotic activity.^26^ A randomized trial of the multi-strain probiotic AB001 suggested a potential dose-dependent association, with modest but statistically significant weight reduction observed among participants receiving higher doses during extended supplementation.^27^ Despite these findings, current literature lacks comprehensive studies that establish generalized conclusions or provide standardized guidelines for precise probiotic dosing.

Adequate strain selection and dosing alone do not guarantee therapeutic efficacy; probiotics must also remain functionally active within the host. Accordingly, probiotic efficacy hinges on two complementary attributes: viability and vitality. Viability refers to survival—the capacity of probiotics to remain alive through processing, storage, and gastrointestinal transit to reach the target site.^28^ Vitality, in contrast, refers to function—the metabolic activity and physiological robustness that enable active interactions with the host. This includes the production of bioactive compounds like bacteriocins and SCFA, which modulate the gut environment and inhibit pathogens.^29-31^ From a translational perspective, strategies that enhance viability are therefore a prerequisite for preserving downstream functional potential. For instance, microencapsulated *Bifidobacterium infantis* demonstrated markedly improved viability during nonrefrigerated storage and enhanced resistance to simulated gastrointestinal stresses compared with non-encapsulated cells.^32^

### 2.3 Resident microbiota

The resident microbiota exhibits substantial inter-individual variability and dynamic changes over time, influenced by factors such as diet, health status, and environment.^33-35^ This shifting landscape profoundly shapes the colonization and efficacy of probiotic interventions, often to a greater extent than host genetic factors.^36^ Consequently, individuals may display highly variable responses to the same probiotic supplementation, as observed in clinical trials where some participants benefit while others experience limited or even adverse effects.^4^^,^^37^

The success of probiotic colonization is largely determined by the existing microbial community, which can either promote or impede the establishment and function of introduced strains.^38^ Moreover, the functional capacity of the resident microbiota shapes how probiotics are metabolized and how their metabolic products impact host physiology. Probiotic strains capable of butyrate production may not confer the expected health benefits when disruption of host SCFA signaling pathways attenuates downstream metabolic and physiological responses.^39^

### 2.4 Pharmacological interventions

Pharmacological interventions can profoundly and bidirectionally influence probiotic colonization and function by altering the gut microbial ecosystem through both direct and indirect mechanisms. Antibiotics (e.g., *β*-lactams, fluoroquinolones) indiscriminately deplete commensal microbiota, disrupting probiotic niches and enabling pathogen overgrowth via competitive release.^40^^,^^41^ Antibiotic-induced microbiota depletion may transiently increase the potential for probiotic engraftment, but actual colonization remains highly individualized and depends on host and baseline microbiome characteristics.^42^

Non-antibiotic drugs also affect probiotic viability and colonization. Non-steroidal anti-inflammatory drugs are known to impair intestinal barrier integrity, and disruption of the mucosal environment may indirectly affect the persistence and functional activity of beneficial microbes.^43^ Importantly, drug–microbiota interactions are not unidirectional; emerging evidence indicates that probiotics can also reciprocally modulate drug efficacy and host responses. For example, *Bifidobacterium animalis* B960 has been reported to enhance the therapeutic efficacy of glibenclamide while alleviating drug-induced adverse effects in a type 2 diabetes model, highlighting a strain-specific probiotic–drug synergy.^44^

### 2.5 Host factors

The efficacy and persistence of probiotics in the gastrointestinal tract are profoundly influenced by a range of host factors that together create a dynamic and selective environment for bacterial engraftment. A fundamental barrier is the harsh physicochemical landscape of the digestive tract, including exposure to gastric acid, bile salts, and digestive enzymes.^45^ The ability of probiotics to survive these challenges is crucial for their subsequent colonization and functional effects in the colon.^46^ Empirical evidence indicates that acid and bile tolerance are critical traits enabling probiotic strains to survive gastrointestinal transit and achieve transient colonization in the human gut.^47^

Beyond physicochemical resilience, host genetic background also influences probiotic engraftment. A prime example is the fucosyltransferase 2 (FUT2) gene, which determines secretor status and directly regulates mucosal and human milk availability of α1,2-fucosylated glycans. FUT2 secretors, through the production of α1,2-fucosylated human milk oligosaccharides, preferentially support the colonization and enrichment of bifidobacterial strains proficient in utilizing these glycans.^48^ Furthermore, host pattern recognition receptor–mediated sensing pathways play a central role in shaping immune responses to probiotics, providing a mechanistic basis through which host genetic variation may contribute to inter-individual differences in immunomodulatory outcomes. Specifically, variations in TLR2 can alter responses to *Lactobacilli* by affecting affinity for lipoteichoic acid, leading to divergent IgA production^49^; likewise, polymorphisms in TLR9 modulate the immune system's reactivity to *Bifidobacterium* DNA, determining the strength of Th1 polarization.^50^

Probiotics exert sex- and age-divergent effects, shaped by a network of interconnected biological variables. At a fundamental level, sex differences in immune system organization establish distinct host contexts for microbial interactions. The foundation for sex-specific responses lies in immune-related gene expression encoded on the X chromosome, including key regulatory genes such as FOXP3, which shape sex-biased immune baselines.^51^ Furthermore, sex hormones modulate both gut microbiota composition and immune responses: estrogen in women enhances microbial diversity, strengthens gut barrier function, and promotes protective taxa such as *Lactobacillus*, which may lower cancer risk.^52^^,^^53^ Such sex-based disparities are dynamic across the lifespan and intersect with age-related physiological declines in sex hormones and altered host physiology. Together with changes in the gut microbiota, these factors may create a host environment less responsive to probiotic interventions in elderly individuals.^54^^,^^55^ Ageing is characterized by immunosenescence and inherent gut microbiota alterations (e.g., reduced diversity), which create a fundamentally different host environment.^56^ Although probiotic supplementation can modify microbial profiles in older adults, functional benefits are often modest and variable, likely reflecting age-related immune dysfunction and altered host–microbe interactions.^57^

Given the multifactorial influences on probiotic efficacy, a universal, one-size-fits-all probiotic solution is unlikely to be effective for all individuals. To address this challenge, there is a growing imperative to develop precise probiotic therapies tailored to specific populations or even individual patients, with recommendations refined down to the strain level. The rapid advancement of multi-omics technologies now provides the necessary tools to comprehensively profile host-microbiome interactions and identify the key determinants of probiotic response. These innovations enable the rational design of personalized probiotic interventions, precisely matched to an individual's unique gut ecosystem and health needs.^58^

## 3 Predictive framework for therapeutic optimization

Building on the mechanistic and ecological determinants of probiotic heterogeneity discussed above, variability in probiotic colonization and therapeutic efficacy can be reframed within the 3PM framework as a challenge of predictive diagnostics based on baseline host–microbiome states. Accumulating evidence demonstrates that probiotic outcomes are not stochastic but can be anticipated from pre-intervention biological signatures.^4^^,^^59^ These predictive diagnostics include microbiome-based markers, such as baseline abundance of closely related taxa and strain-level functional gene clusters (e.g., carbohydrate utilization and bile salt hydrolase genes); host-derived predictors, including FUT2 secretor status and immune receptor polymorphisms (e.g., TLR2 and TLR9); metabolic indicators, such as SCFA production capacity and bile acid transformation profiles; and environmental modifiers, including habitual dietary patterns and concurrent medication exposure. These biomarkers enable stratification of permissive versus resistant microbial ecosystems and probiotic-responsive versus non-responsive individuals, thereby providing a predictive foundation for precision probiotic interventions.^60^

Predictive diagnostics naturally extend toward targeted prevention by aligning microbiome-informed risk stratification with stage-appropriate interventions. Alterations in gut microbial composition and function often precede the onset or exacerbation of metabolic, inflammatory, and immune-mediated disorders, supporting the use of baseline microbiome profiling to identify at-risk individuals and enable early, preventive modulation.^61^^,^^62^ At more advanced stages, predictive frameworks facilitate the selective application of strain-specific probiotic interventions to individuals most likely to respond, thereby mitigating disease progression and reducing inter-individual variability in outcomes.^63^ In established disease contexts, microbiome-informed probiotic strategies may further contribute to prevention by attenuating inflammation, improving treatment tolerance, and reducing recurrence or complication risk.^64^^,^^65^

Within the 3PM framework, predictive and preventive insights ultimately converge on personalized therapy, in which probiotic interventions are tailored to individual host–microbiome contexts rather than applied uniformly. Inter-individual differences in baseline gut microbiota structure, functional capacity, and host physiological traits have been shown to strongly influence probiotic engraftment, persistence, and downstream functional effects.^10^^,^^66^^,^^67^ Accordingly, personalized probiotic therapy emphasizes strain selection and formulation based on ecological compatibility with resident microbial communities, host immune and metabolic phenotypes, and dietary context. Importantly, longitudinal monitoring of microbiome and host responses enables adaptive refinement of interventions, allowing therapeutic strategies to be dynamically adjusted according to individual response trajectories and thereby improving efficacy and durability of outcomes.

**Figure 2.**
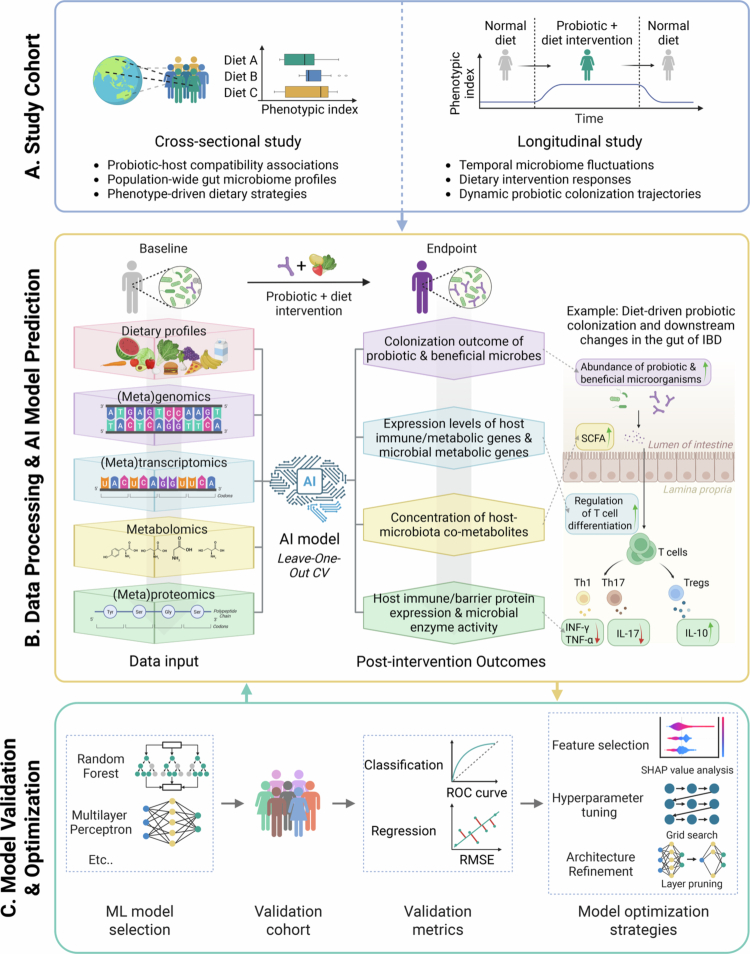
Schematic illustration of the workflow using an AI model to predict probiotic colonization and probiotic-mediated health outcomes under personalized dietary conditions. The workflow comprises three sequential modules: A. Study Cohort module aggregates data from cross-sectional studies (probiotic intervention trials across globally distributed populations with diverse dietary habits) and longitudinal studies (host phenotypic responses to long-term probiotic and dietary interventions). B. The Data Processing & AI Prediction module systematically extracts dietary inputs and multi-omics data (genomics, transcriptomics, proteomics, metabolomics) embedded in the gut flora as training features. An AI model employs leave-one-out validation, leveraging data from all other individuals to predict probiotic treatment efficacy and omics-specific targets (e.g., intestinal probiotic abundance, SCFA levels) for each subject. C. The Validation module involves assessing the generalization capacity of candidate models through classification and regression tasks using an independent validation cohort. Performance was quantified via established validation metrics, including ROC curves for classification tasks and RMSE for regression analyses. Iterative optimization strategies—such as feature selection, hyperparameter tuning and architectural simplification—were implemented to refine model performance. The optimized model will be reapplied to an enlarged training set for iterative refinement, thereby improving predictive accuracy and robustness through enhanced parameter calibration and generalization capacity. *Abbreviations*: ROC (receiver operating characteristic); RMSE (root mean square error).

Based on these principles, effective frameworks require the integration of individual gut microbiota composition and nutrient intake profiles to identify probiotic strains that optimally align with host health outcomes. Beyond optimizing treatment for established conditions, such frameworks hold transformative potential for preemptive health. By enabling early identification of suboptimal microbiota configurations associated with future disease risk, they can guide targeted probiotic and dietary interventions aimed at correcting dysbiotic trajectories before clinical onset, thereby shifting the application paradigm from therapy to prevention. To achieve this, data from two complementary research paradigms—cross-sectional studies and longitudinal studies—must be synergized (Figure 2A).

Cross-sectional studies elucidate probiotic–host compatibility by analyzing gut microbial signatures and dietary patterns (e.g., geographic, age, or lifestyle-specific variations), thereby guiding the development of generalizable, phenotype-driven probiotic strategies. Population-level analyses have revealed strong associations between dietary habits, gut microbiome configurations, and host metabolic responses, providing a foundation for tailoring microbiome-targeted interventions to individual metabolic phenotypes.^68^

In contrast, longitudinal studies unravel the dynamic colonization trajectories of probiotics and their metabolic crosstalk with the host by tracking temporal microbiome fluctuations under dietary interventions (e.g., transitions to plant-based, omnivorous, or high-protein diets). These investigations not only predict short-term metabolic impacts of probiotics but also assess their long-term engraftment stability within evolving gut ecosystems.^69^

By integrating cross-sectional data (reflecting spatial heterogeneity across populations) with longitudinal dynamics (capturing temporal microbial responsiveness), researchers can develop robust AI frameworks to predict probiotic treatment outcomes and host–microbiome multi-omics interactions under dietary interventions (Figure 2B). Baseline inputs may include multi-layered omics profiles—encompassing metagenomics (microbial taxonomy), (meta)transcriptomics (functional gene expression), (meta)proteomics (microbial/host protein activity), metabolomics (host–microbiota co-metabolites), and dietary metadata—to comprehensively map systemic diet–probiotic–host interactions.^70^ These datasets are used to train predictive models with Leave-One-Out Cross-Validation, a rigorous method in which models are trained on data from all but one participant and tested on the excluded individual. AI frameworks can predict endpoint outcomes such as probiotic abundance, host immune gene expression levels, host immune protein expression (e.g., IL-10 cytokine levels), and host immune-metabolic responses (e.g., SCFA concentrations).

These multi-dimensional datasets are used to train AI models (e.g., Random Forest, Neural Networks)^71^^,^^72^ to predict key outcomes, such as probiotic colonization success, host immune markers, and metabolic levels (e.g., SCFAs). To ensure robustness, models are rigorously validated on independent cohorts, with performance quantified using standard metrics for classification (e.g., AUC-ROC) and regression (e.g., RMSE) tasks (Figure 2C).

If model performance generalizes poorly, an iterative optimization cycle is employed. This involves refining the model by selecting the most informative biological features, tuning key parameters, and simplifying the model architecture to enhance predictive power and generalizability across diverse populations.^73^^,^^74^ This continuous feedback loop of training, validation, and optimization ultimately yields a robust tool for forecasting personalized probiotic outcomes.

Currently, research in this field remains at an early stage, with progress limited by technical challenges in multi-omics data integration, the absence of standardized methods for harmonizing heterogeneous datasets, and an incomplete mechanistic understanding of gut bacterial interactions—all of which constrain the predictive power of current models. Nevertheless, recent advances demonstrate promising potential for predicting probiotic colonization and guiding personalized interventions. Clinical studies have shown that patients with specific gut microbial signatures respond more favorably to probiotic treatment for irritable bowel syndrome, indicating that microbiome profiling may forecast therapeutic outcomes.^75^ Computational frameworks have also been developed to predict the colonization success of exogenous bacteria, such as *E. faecium* and *A. muciniphila*, with experimental validation supporting these predictions.^59^ Strain-level tracking following fecal microbiota transplantation further highlights the ability to resolve fine-scale microbial dynamics by integrating pre- and post-intervention microbiome data.^76^

Significant progress has been made in predicting microbiota-associated metabolic outputs and designing individualized dietary strategies. For instance, longitudinal studies have leveraged deep learning to forecast postprandial glycemic responses based on gut microbiome profiles, demonstrating the feasibility of personalized nutrition.^69^ Another deep learning model has successfully inferred food–microbe–metabolite relationships, which enables accurate prediction of post-intervention changes in key metabolites (such as SCFAs and bile acids) based on individual baseline gut microbiome, metabolome data, and dietary intervention strategy.^77^ These advances not only pave the way for precise probiotic therapies tailored to individual microbiome features but also enable the integration of dietary information to deliver personalized nutritional recommendations, heralding a new era of microbiome-informed precision nutrition and therapeutics.

## 4 Method development for precise probiotic mining and engineering

**Figure 3.**
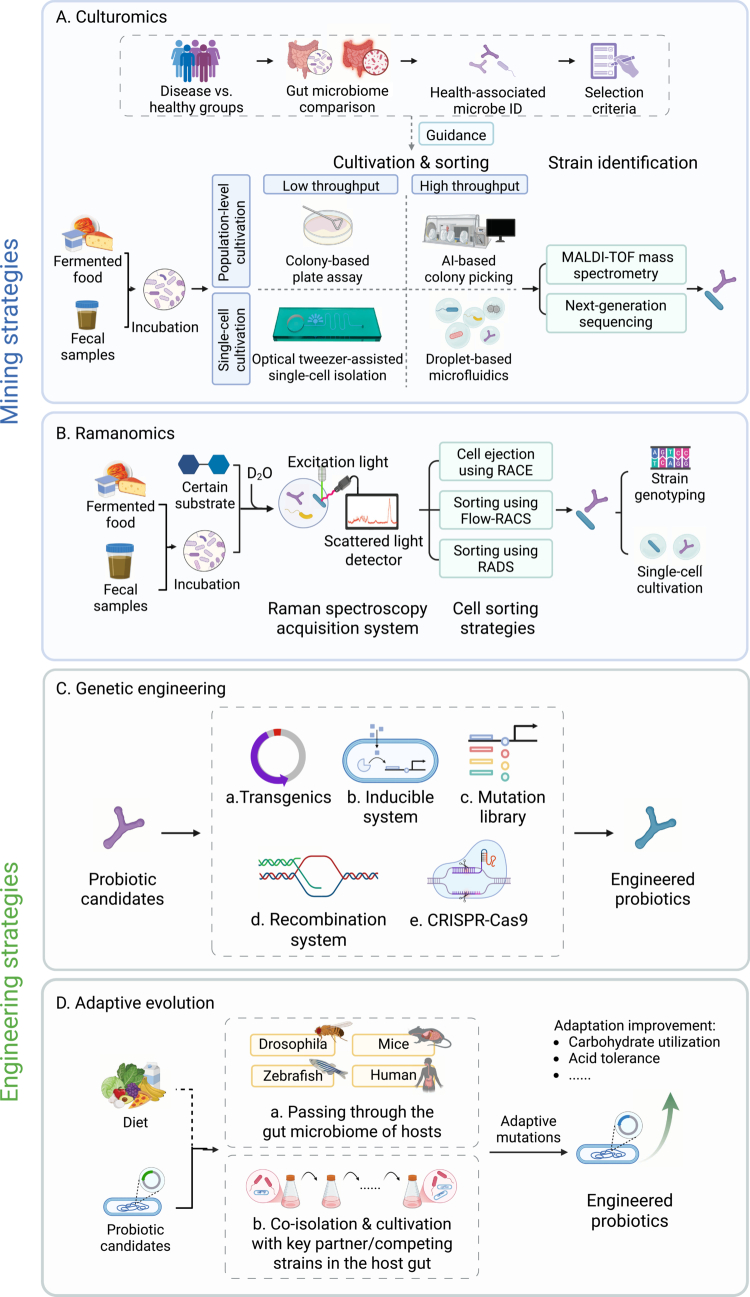
Comprehensive framework for precise probiotic mining and engineering. A. Culturomics-driven discovery: Comparative gut microbiome analysis identifies health-associated taxa, guided by metabolic or antibiotic resistance traits. Combined population-level and single-cell cultivation (e.g., microfluidics) maximizes recovery. Candidate strains are rapidly identified via MALDI-TOF (<1 h), with ambiguous cases resolved by next-generation sequencing for taxonomic and functional validation. B. Ramanomics-enabled sorting: Active microbes are labeled using D₂O and substrate-specific nutrients, detected via Raman spectroscopy (C–D peaks, 2,040–2,300 cm^−1^), and isolated through Raman-activated sorting. Post-sorting, strains undergo genotyping and single-cell culturomics for biobanking. C. Genetic engineering: Transgenics, exogenous molecule-driven inducible systems, mutation libraries, conventional homologous recombination approaches and CRISPR-Cas9-based tools enable precise engineering of candidate strains. D. Adaptive evolution: *In vivo* passage of candidate probiotics through model hosts (*Drosophila*, zebrafish, mice, humans) drives adaptive mutations or enhances strain fitness via synergism with keystone commensals or competitors *in vitro*. *Abbreviations*: MALDI-TOF mass spectrometry (Matrix-assisted laser desorption/ionization time-of-flight mass spectrometry); RACE (Raman-activated cell ejection); Flow-RACS (Flow cytometry-coupled Raman-activated cell sorting); RADS (Raman-activated droplet sorting).

While computational simulations can reveal potential functional characteristics of candidate microbial strains, establishing a prediction-to-acquisition pipeline remains critically dependent on two pivotal challenges: precisely capturing target strains from complex microbial communities to obtain available strain resources and implementing targeted engineering to more effectively regulate the functions of probiotic strains. To address these challenges within the 3PM framework—particularly its personalized intervention pillar—we present an integrated methodological framework for strain development, visually depicted as four subpanels (Figure 3).

### 4.1 Advanced culturomics methods

Culturomics enables the isolation of previously “unculturable” microbes by simulating native ecological niches through tailored culture conditions, combined with multi-omics techniques for precise strain identification and characterization. Target strains for isolation are first identified through comparative microbiome analyses between special populations, such as disease-specific cohorts and their healthy controls, or healthy individuals with long-term consumption of particular foods compared to other dietary cohorts, to uncover differentially abundant or health-associated microorganisms (Figure 3A). Comparative studies, for instance, have revealed a significant reduction of *Faecalibacterium prausnitzii* in patients with Crohn’s disease compared to healthy individuals.^78^ Once target organisms are identified, their phenotypic characteristics can be inferred from metagenome-assembled genomes, as well as proteomic and transcriptomic data.

Rational design of culture conditions, guided by detailed strain information, has significantly advanced probiotic discovery.^79^ By mimicking native ecological niches through media optimization, such as supplementation with rumen fluid, acetate, or mucin, researchers have successfully cultivated fastidious anaerobic gut bacteria.^80^ Traditionally, colony-based plate assays have been commonly used for microbial isolation; however, these methods are frequently labor-intensive and time-consuming. To address these challenges, recent advancements in AI-driven colony picking systems, like CAMII, have automated the selection of isolates by combining colony morphology analysis with genomic data. This integration reduces human bias and greatly speeds up the workflow.^81^ While these approaches enhance efficiency, they are generally limited to population-level evaluations, potentially masking critical single-cell heterogeneity. Innovations such as optical tweezer-assisted single-cell isolation leverage laser-guided precision to extract target single cells from microcultures, though with moderate scalability.^82^^,^^83^ More recently, droplet-based microfluidics platforms have enabled the parallel manipulation of millions of single-cell cultures, facilitating dynamic single-cell behavior analysis and high-throughput screening.^84^^,^^85^ Building on these advances, the AI-driven Digital Colony Picker (DCP) platform automates high-throughput single-cell isolation by integrating deep learning-based phenotypic analysis and laser-induced extraction, enabling precise identification of target strains and parallel cultivation in over ten thousand microchambers.^86-88^

The resulting isolates are subsequently identified using matrix-assisted laser desorption/ionization time-of-flight (MALDI-TOF) mass spectrometry, which rapidly matches their protein spectra to expanding reference databases within one hour.^89^ Isolates unidentifiable by this method are subjected to 16S rRNA gene sequencing; if the sequence similarity to known species is below 98.65%, whole-genome sequencing is performed to determine whether they represent novel probiotic strains.^90^ Although advanced culturomics technologies have rapidly progressed in the past decade, their widespread application to probiotic cultivation is still in its early stages. Future efforts should focus on adapting equipment for anaerobic conditions and improving media and environmental controls to achieve stable, pure cultures of target probiotic strains.

### 4.2 Raman-based approaches for probiotic screening

Raman spectroscopy, an advanced analytical technique, enables rapid, non-destructive, and label-free assessment of the chemical composition of individual cells at single-cell or even subcellular resolution. The technique operates on the principle of Raman scattering: when monochromatic laser light interacts with molecules in a sample, most photons are scattered elastically (Rayleigh scattering), while a small proportion undergoes inelastic scattering, producing a wavelength shift characteristic of the vibrational modes of molecular bonds.^91^ This spectral shift provides a unique molecular fingerprint of the sample, allowing researchers to identify and quantify a wide range of biomolecules simultaneously. Raman spectroscopy allows *in situ* and non-invasive investigation of living microorganisms under physiological conditions. Unlike traditional methods such as fluorescence-activated cell sorting (FACS) or MALDI-TOF mass spectrometry—which often require fluorescent labeling, destructive sample preparation, or population-level averaging—Raman spectroscopy preserves cellular viability while offering single-cell resolution and direct metabolic profiling. These unique advantages make it a powerful tool for studying microbial physiology, metabolic activity, and ecological interactions within complex communities.

Building on these strengths, Ramanomics, an extension of the “Ramanome” concept described by Teng et al.^92^ refers to the systematic, high-throughput phenotyping of microbial cells by collecting and analyzing single-cell Raman spectra (SCRS) from complex communities. This enables comprehensive and quantitative characterization of metabolic states, stress responses, and phenotypic diversity at single-cell resolution. As illustrated in the workflow (Figure 3B), microbial samples are first collected from sources such as fermented foods or feces and incubated with a stable isotope probe (SIP), such as labeled substrate (13C-glucose, 15NH4+) or heavy water (D₂O).^93^ Compared to substrate-specific approaches, D₂O SIP universally labels all metabolically active cells without requiring prior knowledge of substrate utilization, does not disturb the natural substrate pool, and is cost-effective.^94^^,^^95^ D₂O incorporation is readily detected by Raman spectroscopy: deuterium can be rapidly assimilated into cellular macromolecules,^96^^,^^97^ resulting in the formation of C-D bonds that shift the characteristic C─H peak at 2,800-3,100 cm^−1^ into the silent region (2,040−2,300 cm^−1^), providing a sensitive and specific marker for metabolic activity.^98-100^

After incubation, individual cells are analyzed by the Raman spectroscopy acquisition system, generating the Ramanome for the community. Cells displaying metabolic activity, such as those exhibiting a prominent C–D peak, can then be isolated by advanced Raman-activated cell sorting strategies—including Raman-activated cell ejection (RACE),^101^^,^^102^ Raman-activated cell sorting (RACS, as well as Flow-RACS, flow cytometry-coupled RACS),^94^^,^^100^^,^^103^^,^^104^ and Raman-activated droplet sorting (RADS).^105^^,^^106^ Post-sorting workflows bifurcate into two synergistic paths: (1) Single-cell sequencing enables taxonomic resolution and functional annotation of sorted cells, directly linking *in situ* activity (e.g., C–D peak profiles) to genetic potential for novel probiotic discovery.^82^^,^^83^ (2) Single-cell cultivation can be seamlessly integrated with single-cell cultivation platforms to achieve massively parallelized microdroplet culture of functionally validated probiotic candidates.^86-88^

The application of Ramanomics, combined with advanced data analytics, has revolutionized the rapid and accurate identification and classification of probiotic bacteria. Recent studies have showcased the remarkable capability of machine learning models trained on SCRS data, achieving up to 97.3% accuracy in distinguishing closely related Lactobacillus species and subspecies. This underscores the potential of utilizing Raman spectroscopy for high-resolution taxonomic profiling within complex probiotic formulations.^107^ Moreover, the integration of RACS with D₂O-based SIP has proven successful in isolating metabolically active mucin-utilizing bacteria from the murine gut microbiome. These functionally sorted strains were subsequently assembled into a precise probiotic consortium that effectively suppressed colonization by the enteric pathogen *C. difficile* in mice.^94^^,^^100^ Additionally, the SCIVVS workflow, which integrates SCRS-based identification, *in situ* viability and vitality assessment, and genome-based source tracking at single-cell resolution, has been validated for the swift quality control of commercial probiotic products.^108^ This culture-free approach enables comprehensive live-cell enumeration, species-resolved vitality profiling, and high-coverage genomic characterization within hours, presenting significant enhancements in speed, sensitivity, and automation compared to traditional methodologies.

### 4.3 Genetic engineering approaches

Advancements in synthetic biology have broadened the genetic engineering toolkit, facilitating the therapeutic enhancement of probiotics through functionalization. Illustrated in Figure 3C, five fundamental strategies—transgenics, inducible systems, mutation libraries, recombination systems, and CRISPR-Cas9—combine to establish the groundwork for the strategic engineering of probiotics, with each method providing specific mechanisms and individual advantages.

Transgenic approaches involve the stable introduction and expression of exogenous genes, typically via plasmid-based systems, to confer entirely new metabolic or therapeutic functions on probiotics. For example, *Escherichia coli* Nissle 1917 (EcN) has been widely used as a genetically tractable chassis for engineered live bacterial therapeutics, enabling the delivery of therapeutic functions for gastrointestinal and inflammatory diseases.^109^^,^^110^

Inducible expression systems provide an additional layer of control, allowing gene expression to be precisely regulated in response to specific environmental or chemical cues. Various inducible promoters—including rhamnose-, mannan-, bile acid-, and IPTG-inducible systems—have been functionally characterized in Bacteroides species, enabling tunable and context-dependent gene expression for therapeutic delivery or biosensing applications.^111-113^ Similarly, the nisin-controlled expression (NICE) system in *Lactococcus lactis*, together with other quorum-sensing and chemically inducible promoters, has been extensively characterized and implemented in lactic acid bacteria, enabling tightly regulated and context-responsive gene expression for therapeutic and biotechnological applications.^114^^,^^115^

Mutation library strategies, such as transposon mutagenesis, enable the systematic dissection of gene function, unravel complex genetic networks, and facilitate the high-throughput generation and screening of diverse mutant populations.^116^ These approaches have been applied to *Bacteroides*, *Lactobacillus*, *Bifidobacterium*, and other gut commensals to identify genes essential for fitness, metabolism, colonization, and host interaction.^117-119^

Recombination-based methods, including homologous recombination and recombineering, enable precise and targeted genetic modifications such as gene deletions and insertions. Markerless gene knockouts and multiplex genome modifications have been achieved in gut commensals using Cre-lox systems,^120^ and high-throughput platforms such as multiplex automated genome engineering (MAGE) and serine recombinase-assisted genome engineering (SAGE).^121^ MAGE uses synthetic single-stranded DNA and phage-derived annealing proteins to introduce multiplexed genomic edits during replication, and has been successfully applied to optimize metabolic pathways in *Escherichia coli*, providing a foundation for extending recombineering-based genome engineering to other bacterial systems.^122^ SAGE utilizes a serine recombinase to integrate cargo DNA at specific chromosomal sites, followed by excision of selection markers, enabling efficient insertion of up to ten genes in non-model gut bacteria such as *Bacteroides* and *Prevotella.*^123^

CRISPR/Cas9, an RNA-guided endonuclease tool, enables precise genome editing by inducing targeted DNA double-strand breaks and leveraging cellular repair mechanisms.^124^ The advent of CRISPR-Cas9 genome editing has revolutionized the field by providing a highly programmable, efficient, and broadly transferable platform for both gene disruption and precise sequence modification.^125^ Both CRISPR-Cas9 and CRISPR interference (CRISPRi) have been implemented in lactic acid bacteria, *Bacteroides*, and *Escherichia coli* Nissle 1917 to modulate key genetic loci, enhancing therapeutic function and enabling biocontainment strategies.^126-130^

### 4.4 Adaptive evolution strategies for genetic engineering

Adaptive evolution refers to the process by which microbial populations acquire genetic changes, primarily single-nucleotide variants, insertions/deletions, and, less frequently, structural variants, that enhance their fitness within specific environments.^131^ In host-associated microbiomes, adaptive evolution is prevalent and driven by selective pressures, including diet, antibiotics, host immunity, and inter-microbial competition.^132^^,^^133^ Notably, adaptive evolution is characterized by its rapid timescale and specificity to individual hosts and exposures.^134^^,^^135^ Recent metagenomic studies have demonstrated that adaptive mutations can emerge and spread within human gut microbial populations over remarkably short periods, often in response to personalized factors such as diet or antibiotic use.^136^ The principal advantage of adaptive evolution lies in its capacity to generate strain-level diversity and functional innovation, enabling microbes to exploit new ecological niches, enhance colonization efficiency, and adapt to host-imposed challenges.^137^^,^^138^ These genetic and functional adaptations not only refine probiotic traits but also profoundly impact host health, disease susceptibility, and the success of microbiome-targeted therapies.^139-142^

Two principal adaptive evolution strategies have been developed to enhance the fitness and functional performance of probiotic candidates (Figure 3D). The first one involves serial passage of probiotic strains through the gut luminal environment of hosts, thereby exposing them to complex and host-specific selective pressures such as diet, indigenous microbiota, and host physiology^133^ and thus gaining fitness advantages for them. For example, *L. plantarum* HNU082 was passed through the gastrointestinal tracts of humans, mice, and zebrafish, consistently acquiring highly similar single-nucleotide mutations across hosts, primarily affecting carbohydrate utilization and acid tolerance, which resulted in markedly enhanced colonization and ecological competitiveness.^143^ Likewise, *L. plantarum* NIZO2877, after consecutive passages in the gut of *Drosophila melanogaster*, rapidly fixed mutations in the *ackA* gene, significantly improving its growth-promoting effects and fitness under nutrient-poor conditions.^144^ In another study, EcN evolved in the mouse gut under varying microbiota complexity and dietary regimes, accumulating mutations related to carbon metabolism, stress response, and adhesion, ultimately increasing its competitive fitness and colonization in low-diversity environments.^139^ These pre-adapted strains can be isolated, purified from stool samples and reintroduced into compatible hosts, prolonging microbial colonization and further enhancing therapeutic efficacy.

The second strategy centers on co-isolation and co-cultivation of probiotic candidates with key partner or competing strains from the host gut, facilitating the selection of variants with superior ecological fitness through direct microbial interactions. Notably, *F. prausnitzii* was co-isolated and gradually adapted with its partner *Desulfovibrio piger* from healthy human fecal samples.^145^ Using a stepwise adaptation protocol, researchers obtained *F. prausnitzii* variants tolerant to oxygen exposure; when co-cultured with *D. piger*, these oxygen-adapted strains exhibited a dramatic increase in yield, demonstrating the effectiveness of co-cultivation approaches for enhancing the robustness and scalability of next-generation probiotics.

A notable advantage of adaptive evolution strategies over synthetic biology approaches is their inherent alignment with natural selection processes, which often translates to enhanced safety profiles. Unlike genetically engineered probiotics—which may involve artificial gene insertions, CRISPR-Cas9 modifications, or transgenic elements—adaptive evolution relies on incremental, context-specific mutations that arise under biologically relevant pressures. This minimizes risks associated with horizontal gene transfer, unintended metabolic disruptions, or ecological imbalances, as the evolved traits are inherently compatible with the host-microbiome environment. Furthermore, adaptive evolution circumvents regulatory and public skepticism tied to genetically modified organisms (GMOs), as the resulting strains are not classified as “engineered” but rather as naturally optimized variants. While synthetic biology offers precision in introducing targeted functions, adaptive evolution provides a safer, ecologically harmonized pathway for probiotic development, particularly for applications requiring long-term colonization or deployment in sensitive populations. However, this approach may lag in speed and specificity compared to cutting-edge engineering tools, highlighting the need for complementary use of both strategies in advancing precise probiotics.

## 5 Future outlook of precise probiotic therapy

### 5.1 Emerging innovations in probiotic engineering

The advent of high-throughput multi-omics technologies has revolutionized our understanding of the gut microbiome and its interactions with the host. When coupled with advances in artificial intelligence and machine learning, these datasets enable the development of predictive models that can forecast individual responses to probiotic interventions with unprecedented accuracy. The integration of multi-layered omics data allows for the identification of key microbial signatures, metabolic pathways, and host factors that determine the efficacy of probiotic engraftment. However, realizing the full potential of precise probiotics requires overcoming significant computational and technical hurdles, including the harmonization of heterogeneous datasets, the standardization of data processing pipelines, and the translation of predictive biomarkers into clinically actionable insights.

Synthetic biology and genome engineering are catalyzing the development of designer probiotics with enhanced stability, targeted metabolic functions, and customizable therapeutic properties. Techniques such as CRISPR-Cas9 genome editing, inducible expression systems, and adaptive laboratory evolution enable precise modification of probiotic strains to optimize colonization potential, metabolic output, and interaction with the host immune system. For example, engineered strains can be programmed to produce specific bioactive compounds, degrade toxic metabolites, or deliver therapeutic molecules directly within the gut. Despite these advances, the safety, long-term stability, and ecological impact of genetically modified probiotics remain major concerns. The risk of horizontal gene transfer, unintended off-target effects, and disruption of native microbial communities must be rigorously assessed through preclinical and clinical studies. Regulatory frameworks will need to adapt to ensure that engineered probiotics meet high standards for safety, efficacy, and traceability before their widespread clinical adoption.

### 5.2 Comparative strain-specific functions of *Lactobacillus* and *Bifidobacterium* spp. and their clinical translation

Although probiotic efficacy is ultimately determined at the strain level, *Lactobacillus* and *Bifidobacterium* spp.—the two most widely applied probiotic genera—exhibit distinct functional orientations that are highly relevant for clinical translation. These differences arise from pronounced heterogeneity in physiological tolerance, adhesion capacity, metabolic activity, and immunomodulatory potential, which collectively shape probiotic survival and function *in vivo*.

Even fundamental survival traits display marked strain dependence. Characterization of lactic acid bacteria and bifidobacteria has revealed striking intra-species divergence in resistance to gastrointestinal stressors. For example, while *Lactobacillus rhamnosus* LB-3 VK6 and *L. delbrueckii* LE VK8 show broad antibiotic resistance**,** *L. plantarum* LM VK7 is highly sensitive to gastric juice, ceasing growth at low concentrations, whereas *L. acidophilus* IMV B-7279 and *Bifidobacterium animalis* VKL/VKB remain viable even under extreme gastric conditions.^146^ Beyond survival, adhesion capacity further stratifies probiotic action: highly adhesive strains such as *L. casei* IMV B-7280 and *B. animalis* VKL/VKB are predisposed to sustained mucosal interaction, whereas low-adhesion strains exert more transient luminal effects, enabling precision matching of strains to upper gastrointestinal or barrier-restorative applications.^147^

These strain-level properties converge into genus-level functional polarization. *Lactobacillus* strains more frequently promote mucosal immune activation, including enhanced IgA secretion and Th1/Th17-associated responses, supporting applications in infection prevention and vaccine adjuvanticity.^148^ In contrast, *Bifidobacterium spp.* preferentially induce tolerogenic and anti-inflammatory pathways, such as regulatory T-cell expansion and suppression of pro-inflammatory cytokines, which align with therapeutic goals in chronic inflammatory and metabolic disorders.^149^

This functional dichotomy is reflected in clinical use, where *Lactobacillus rhamnosus* GG is effective in preventing pediatric diarrhea, whereas *L plantarum* 299v is primarily applied to improve gastrointestinal function and alleviate symptoms of IBS.^150^^,^^151^ Clinical studies have shown that supplementation with *B. longum* 1714 strain can reduce symptoms of depression, linking gut health to mental health via the gut-brain axis.^152^ Additionally, *B. salivarius UBL S22* has been shown to improve metabolic outcomes, such as lowering blood cholesterol levels and enhancing insulin sensitivity in individuals with metabolic syndrome.^153^

Collectively, these examples underscore a shift from genus- or species-based classification toward a gene-to-function-to-phenotype framework, enabling mechanism-driven, phenotype-guided probiotic selection within the 3PM paradigm. This paradigm emphasizes the importance of not only selecting strains based on their metabolic and immunological profiles but also considering genetic predisposition and existing microbiota composition.

### 5.3 Translational implications: microbiome modulation

Recent advancements in microbiome research emphasize the potential for personalized microbiome interventions, where the selection of probiotics and prebiotics is guided by an individual’s microbiome composition and phenotype. The aim is to optimize treatment strategies based on the unique microbiota of each individual, fostering a more effective, personalized approach to microbiome modulation. Personalized probiotic and prebiotic selection is central to the evolving paradigm of precision nutrition. Through a deeper understanding of microbiome composition, interventions can be tailored to selectively stimulate the growth of health-promoting microbiota. This approach goes beyond the broad application of conventional prebiotics, offering more targeted interventions that align with specific host needs.

Emerging postbiotic and nanobiotic strategies are gaining traction as cutting-edge, microbiome-modulating approaches. Postbiotics—the metabolic byproducts produced by probiotics—are proving to have significant health benefits. For example, SCFAs, produced during the fermentation of dietary fibers, play a critical role in maintaining gut health, enhancing immune function, and reducing inflammation.^154^

Nanobiotics, particularly nanoceria-based strategies, are also revolutionizing microbiome-based therapies. Recent studies demonstrate that cerium oxide nanoparticles (CeO2) can modulate gut microbiota and reduce cholesterol levels in obese animal models.^155^ This innovative approach combines the benefits of prebiotics with the added potential of nanotechnology, creating an entirely new dimension of microbiome modulation. In clinical studies, nanoceria's ability to act as a prebiotic has been shown to enhance gut microbial diversity and improve metabolic profiles, highlighting its potential in the prevention and management of metabolic disorders like obesity and atherosclerosis.^5^

### 5.4 Understanding the role of gut microbiota in systemic conditions

As the role of gut microbiota in health management becomes increasingly prominent, its influence on systemic conditions beyond the gastrointestinal tract—particularly infertility and the gut–liver–gonadal axis—has attracted increasing attention.^156^^,^^157^ Accumulating evidence indicates that gut microbiota contributes to reproductive health by modulating hormonal homeostasis, immune tone, and host metabolic pathways. Dysbiosis has been associated with impaired sex hormone regulation, chronic inflammation, and reproductive disorders such as infertility, polycystic ovary syndrome, and endometriosis, underscoring the importance of gut–reproductive crosstalk in systemic physiology.^157^^,^^158^

Mechanistically, the gut microbiome interacts with the hypothalamic–pituitary–gonadal axis and participates in sex steroid metabolism, thereby influencing ovulation, menstrual cyclicity, and spermatogenesis.^159^ Recent genetic epidemiology and clinical studies further support a causal link between specific gut microbial taxa and infertility-related outcomes, including altered progesterone metabolism and impaired embryo implantation, providing direct evidence that microbial activity can shape reproductive physiology.^160^

Beyond reproductive health, the implications of microbiota-based interventions extend to a broad range of systemic diseases. Growing evidence supports a contributory role of gut microbiota in autoimmune disorders, cardiovascular disease, and chronic kidney disease through immune modulation, metabolic signaling, and regulation of systemic inflammation.^161-164^ Consequently, multicenter, stratified 3PM-oriented studies and longitudinal registries are urgently needed to validate microbiota-based interventions across diverse populations and disease contexts.^165^^,^^166^ Such efforts will be critical to establish causal relationships, refine stratified intervention strategies, and facilitate the clinical translation of microbiome-modulating therapies for systemic diseases spanning reproductive, immune, cardiovascular, and renal systems.

### 5.5 Cost-benefit considerations of personalized probiotic therapies

As personalized probiotic therapies gain momentum, it is crucial to consider not only their potential for enhanced therapeutic outcomes but also the practical and economic implications of their widespread adoption. While tailoring probiotic interventions to individual microbiome profiles offers the promise of higher efficacy and more precise treatment, it also comes with increased costs. Profiling patients to determine the most suitable probiotics requires additional diagnostic tests, which extend the time and costs of treatment. Moreover, as the patient population is stratified into smaller subgroups, the cost of research and development must be recouped from a smaller market, potentially making these treatments more expensive for patients.

This cost issue is particularly significant when considering the application of personalized probiotics in both chronic and acute conditions. For chronic, complex diseases such as Crohn's disease or ulcerative colitis, the higher costs associated with personalized treatments may be justified due to the potential for improved long-term outcomes. However, for more transient, self-limiting conditions like traveler’s diarrhea or the common cold, the additional costs may outweigh the benefits, as the therapeutic window is often too short to justify extensive patient profiling and personalized intervention.

Thus, while the future of personalized probiotic therapies looks promising, it is essential to strike a balance between the promise of precision medicine and the economic realities of implementation. The cost-benefit analysis will likely be more favorable for chronic, severe conditions where personalized treatments can offer significant improvements in outcomes. For less severe conditions, a more cost-effective, generalized approach may remain the preferred solution, and personalized therapies may need to be selectively applied to those most likely to benefit.

### 5.6 Limitations of microbiota characterization

Microbiota profiling is central to personalized probiotic therapies, but it is important to acknowledge the limitations of current methods. Non-intrinsic factors, such as transit time, specific diet consumption (e.g., wine), and environmental pollutants, can cause significant fluctuations in microbiota composition, often reflecting transient states rather than the host’s stable microbiome.^167-169^ Consequently, stool samples may not fully represent the long-term microbiota, which can affect the reliability of predictive models.^170^

Additionally, stool-based analyses mainly reflect the microbiota in the distal colon lumen, which may not accurately represent the microbiota attached to the mucosal lining or present in the small intestine.^171^ While sampling from these areas is possible, it is more invasive and impractical for routine use. This means that current methods may miss important microbial communities and fail to capture the full complexity of the gut microbiome.

Given these challenges, predictive models must consider the variability and limitations in microbiota data. The accuracy of these models depends on the quality of the data, and if the data is influenced by short-term factors, important signals may be missed, leading to inaccurate recommendations. Thus, understanding these limitations is crucial for developing effective, real-world predictive frameworks for microbiota-based therapies.

Looking forward, the ultimate goal is to establish a holistic paradigm for microbiome-based therapy that transcends single-strain or even multi-strain probiotic formulations. This paradigm is fundamentally aligned with the 3PM framework. Future strategies will likely integrate precision-designed probiotics, personalized dietary interventions, prebiotic and postbiotic components, and even engineered native microbes into comprehensive therapeutic regimens. These interventions will be guided by predictive stratification based on dynamic, longitudinal multi-omics monitoring of the host-microbiome ecosystem, enabling targeted prevention for at-risk individuals and adaptive, individualized interventions informed by real-time feedback. The development of digital health platforms and wearable biosensors will further facilitate this 3PM-informed, individualized management, empowering patients and clinicians to co-create tailored health solutions. As interdisciplinary collaborations deepen, the vision of precision microbiome therapy—seamlessly embedding predictive diagnostics, targeted prevention, and personalized care into a systems biology approach—will become an attainable reality, fundamentally transforming the prevention, diagnosis, and treatment of a wide range of diseases.
